# Adiponectin Protects Rat Myocardium against Chronic Intermittent Hypoxia-Induced Injury via Inhibition of Endoplasmic Reticulum Stress

**DOI:** 10.1371/journal.pone.0094545

**Published:** 2014-04-09

**Authors:** Wenxiao Ding, Xiaofeng Zhang, Hanpeng Huang, Ning Ding, Shijiang Zhang, Sean Z. Hutchinson, Xilong Zhang

**Affiliations:** 1 Department of Respirology, The First Affiliated Hospital with Nanjing Medical University, Nanjing, China; 2 Department of Cardiothoracic Surgery, The First Affiliated Hospital with Nanjing Medical University, Nanjing, China; 3 Morsani College of Medicine, Tampa, Florida, United States of America; Virginia Commonwealth University, United States of America

## Abstract

Obstructive sleep apnea syndrome (OSAS) is associated with many cardiovascular disorders such as heart failure, hypertension, atherosclerosis, and arrhythmia and so on. Of the many associated factors, chronic intermittent hypoxia (CIH) in particular is the primary player in OSAS. To assess the effects of CIH on cardiac function secondary to OSAS, we established a model to study the effects of CIH on Wistar rats. Specifically, we examined the possible underlying cellular mechanisms of hypoxic tissue damage and the possible protective role of adiponectin against hypoxic insults. In the first treatment group, rats were exposed to CIH conditions (nadir O2, 5–6%) for 8 hours/day, for 5 weeks. Subsequent CIH-induced cardiac dysfunction was measured by echocardiograph. Compared with the normal control (NC) group, rats in the CIH-exposed group experienced elevated levels of left ventricular end-systolic dimension and left ventricular end-systolic volume and depressed levels of left ventricular ejection fraction and left ventricular fractional shortening (p<0.05). However, when adiponectin (Ad) was added in CIH + Ad group, we saw a rescue in the elevations of the aforementioned left ventricular function (p<0.05). To assess critical cardiac injury, we detected myocardial apoptosis by Terminal deoxynucleotidyl transfer-mediated dUTP nick end-labeling (TUNEL) analysis. It was showed that the apoptosis percentage in CIH group (2.948%) was significantly higher than that in NC group (0.4167%) and CIH + Ad group (1.219%) (p<0.05). Protein expressions of cleaved caspase-3, cleaved caspase-9, and cleaved-caspase-12 validated our TUNEL results (p<0.05). Mechanistically, our results demonstrated that the proteins expressed with endoplasmic reticulum stress and the expression of reactive oxygen species (ROS) were significantly elevated under CIH conditions, whereas Ad supplementation partially decreased them. Overall, our results suggested that Ad augmentation could improve CIH-induced left ventricular dysfunction and associated myocardial apoptosis by inhibition of ROS-dependent ER stress.

## Introduction

Obstructive sleep apnea syndrome (OSAS) is a common disease characterized by repetitive episodes of complete or partial upper airway occlusion during sleep, leading to intermittent hypoxemia, frequent arousals, daytime fatigue and negative intrathoracic pressure. It has been reported that OSAS is associated with many cardiovascular disorders, such as heart failure [1], hypertension [2], atherosclerosis [3], arrhythmia [4], and higher cardiovascular mortality and morbidity rates [5], [6]. However, the exact mechanisms of poor cardiovascular outcomes resulting from OSAS remain unknown. To explain a mechanism of injury, our lab has investigated the association that adiponectin (Ad) has with OSAS.

Adiponectin, a circulating cytokine derived from white adipose tissue and cardiomyocyte [7], [8], has been suggested to possess cardioprotective properties, as an anti-inflammatory, anti-atherogenic, anti-hypertensive and insulin sensitizing agent [9], [10], [11], [12]. Pischon et al's landmark study showed that high plasma Ad levels correlated to a lower incidence of coronary heart disease in healthy participants [13]. By comparison, other studies have shown that plasma Ad level decreased in obese people [14], [15] and patients with diabetes mellitus [15], hypertension [16], insulin-resistance [17] and coronary artery disease [18], [19]. Additionally, in vivo studies have demonstrated that deficiency of Ad exacerbates cardiac damage under various pressure overload states [20], [21]. In human trials, it has been observed that the plasma Ad level was decreased in OSAS patients compared to healthy controls [22], [23]. We believe the decrease in plasma/serum Ad levels within chronically hypoxic OSAS patients is a direct result of cell oxidative stress.

Endoplasmic Reticulum (ER) stress has been implicated as a major factor in cardiovascular etiologies, such as cardiac hypertrophy and failure, atherosclerosis and ischemic heart disease [24], [25], [26]. Yun-fei Bian et al made the important finding that Ad provided the cardiomyocytes with partial protection from hypoxia/reoxygenation induced injury by inhibiting ER stress [27]. However, no clear relationships have been elucidated among chronic intermittent hypoxia (CIH), ER stress and Ad *in vivo*. In this study, we designed a chronic intermittent hypoxia model to study the possible interactions among these factors.

## Materials and Methods

### Antibodies and reagents

The 78-kDa glucose-regulated protein (GRP78), CCAAT/enhancer-binding protein-homologous protein/growth arrest and DNA damage-inducible gene 153 (CHOP), PKR-like ER kinase (PERK), phospho-PERK, eukaryotic translation initiation factor 2α (eIF2α), phospho-eIF2α, c-Jun N-terminal kinase (JNK1/2), phospho-JNK1/2, p38 mitogen-activated protein kinase (MAPK), phospho-P38 MAPK, caspase-12, caspase-9, caspase-3 and β-actin were purchased from Cell Signaling Technology (Danvers, MA, USA). The inositol-requiring enzyme 1 (IRE1), phospho-IRE1, splicing of X-box binding protein 1 (XBP-1(s)) and activating transcription factor 6 (ATF6) were purchased from Abcam Ltd (USA). Ad was purchased from BioVision (USA) (rat globular adiponectin, diluted in PBS, free from endotoxins). Unless specially stated, all chemical reagents were purchased from Sigma (St. Louis, MO, U.S.A).

### Animals

A total of 45 male Wistar rats, weight 200–230 g at the beginning of the experiments, 8 weeks of age were purchased from Shanghai Silake Ltd.Inc. The rats (specific pathogen free) were housed in Animal Care Center under a 12-hour light-dark cycle with 24°C and allowed free access to standard chow and water. This study was approved by the Animal Ethic Committee of Nanjing Medical University.

The protocol for the CIH exposure has been reported previously [28], [29]. The rats were randomly divided into three groups: normal control (NC) group, chronic intermittent hypoxia (CIH) group, chronic intermittent hypoxia and adiponectin supplement (CIH + Ad) group. The rats were housed in the regular cage with a gas control delivery system, which regulated the flow of air, nitrogen, and oxygen into cages. A series of programmable solenoids and flow regulators decreased the inspired oxygen fraction (Fi,o_2_) from ∼21% to ∼5–6% and sustained for 15∼20 s over a 1 min period with a rapid reoxygenation to room air levels in the subsequent 1 min period. Intermittent hypoxia events in CIH group and CIH + Ad group were administered from 9 am to 5 pm and persisted 35 consecutive days. NC group received the gas-flow exposure as CIH group and CIH + Ad group, but using only room air. Rats in CIH + Ad group were also treated with the intravenous injection of Ad at the dosage of 10 μg per time, twice a week for successive 5 weeks. A simultaneous injection of saline (0.5 ml per time) was carried out in NC group and CIH group. Data were collected at the end of 5^th^ week (day 35).

### Echocardiography

On day 35 of the experiment, the rats was anesthetized by using 1–2% isoflurane, transthoracic echocardiography was performed before the rats were killed. We measured the short axis M- and B-mode images of the left ventricular using the machine of VisualSonics (Canada) with a 20-MHz linear transducer. Heart rate (HR), left ventricular end-diastolic dimension (LVDd, in mm), left ventricular end-systolic dimension (LVDs, in mm) was obtained to calculate the left ventricular end-systolic volume (LVESV; 7.0/(2.4+ LVDs)*LVDs^3^) and left ventricular end-diastolic volume (LVEDV; 7.0/(2.4+ LVDd)*LVDd^3^), left ventricular ejection fraction (LVEF; 100%*(LVEDV - LVESV)/LVEDV), left ventricular fractional shortening (LVFS; 100%*(LVDd - LVDs)/LVDd).

### Blood sample and Tissue processing

After echocardiography, the rats were anesthetized by using pentobarbital. Then the chest was opened for collecting blood and heart tissue. The blood was centrifuged at 3,000×g at 4°C for 15 min and the serum was obtained and stored at −70°C until ready for analysis. The heart tissue was quickly isolated and part was infused into 4% paraformaldehyde while part was stored at −70°C quickly. After 48 h of infusion of 4% paraformaldehyde, the tissues were paraffinembedded. Myocardial tissue sections were used for histological analysis.

### Measurement of serum Ad

The concentration of serum Ad was detected by radioimmunoassay. Samples were determined following the instruction of the kit (R&D, USA) in triplicate.

### Quantitative real-time RT-PCR analysis

Total RNA was isolated from left ventricular tissue homogenates of heart by using the TRIzol reagent (Invitrogen, USA) according to the manufacture's specifications. 1 μg of total RNA was reversely transcribed to complementary DNA (cDNA) using Transcriptor First Strand cDNA Synthesis Kit (Roche, Germany). Real-time QPCR was performed by using Power SYBR Green QPCR Master Mix (Applied Biosystems, Foster City, California, USA) and PCR primers (Invitrogen, USA) for rat ER stress-related genes: GRP78 (forward (5′- GCAGTTGCTCACGTGTCTTG-3′); reverse (5′- TCCAAGGTGAACACACACCC-3′)), CHOP (forward (5′- CGCATGAAGGAGAAGGAGCA-3′); reverse (5′- TGTGGTCTCTACCTCCCTGG-3′))and β-actin (forward (5′- CAGGGTGTGATGGTGGGTATGG-3′); reverse (5′- AGTTGGTGACAATGCCGTGTTC-3′)). Cycling parameters were as follows: 95°C, 10 minutes and 40 cycles of 95°C for 15 seconds and 60°C for 1 minute. The dissociation curves were performed to verify that a single product was obtained. All PCR assays were performed in triplicate. The PCR fluorescent signals for GRP78 and CHOP were standardized to PCR fluorescent signals obtained from an endogenous reference (β-actin). Comparative and relative quantifications of these gene products normalized to β-actin and the control group were calculated by the 2^−△△Ct^ method.

### Western blot analysis

Myocardial tissue was homogenized with Tissue Protein Extraction Reagent (Thermo scientific, USA) containing 1 mM of PMSF and phosphatase inhibitor cocktail (Roche, Germany) according to the instruction. Then heart homogenates were centrifuged at 10000×g for 5 minutes and the supernatants were collected. The bicinchoninic acid method was used to detect the protein concentration by using the protein assay kit (Thermo Scientific, Rockford, USA). Total heart lysates were used to quantify proteins mentioned above by western blot. Equal protein amounts (30 μg) of cell lysates were subjected to electrophoresis on 10% sodium dodecyl sulfate PAGE and transferred to polyvinylidene fluoride membranes. The membranes were blotted with 5% bovine serum albumin in TBS with 0.1% Tween-20 at pH 7.6 for 1 h at room temperature and incubated with primary antibodies diluted in 5% bovine serum albumin in TBS with 0.1% Tween-20 at pH 7.6 at 4°C for one night with gentle shaking, followed by incubation with a peroxidase-labeled secondary antibody diluted in 5% bovine serum albumin in TBS with 0.1% Tween-20 at pH 7.6 for 1 h at 37°C. The blots were detected using enhanced ECL kit (Thermo Scientific, Rockford, USA) and then exposed by using the digital imaging system (Molecular Imager ChemiDocTM XRS+ System), which offers sensitive chemiluminescent detection (Bio-Rad Laboratories Inc, Hercules, CA, USA). The intensity of each band was normalized to β-actin analyzed using Image Lab 2.0 Software (Bio-Rad Laboratories Inc, CA, USA).

### TUNEL staining

We carried out terminal deoxynucleotidyl transfer-mediated dUTP nick end-labeling (TUNEL) by use of *In Situ* Cell Death Detection Kit, POD (Roche, Germany), according to the manufacturer's instructions. Briefly, after deparaffinization and rehydration, the sections were treated with protease K at a concentration of 20 μg/ml for 15 minutes. The slides were immersed in TUNEL reaction mixture for 60 min at 37°C in a humidified atmosphere in the dark. Then DAPI was used to incubate the slides for 5 min to show the characteristic blue nuclear staining. The slides were analyzed by fluorescence microscope. Then the obtained images were merged and analyzed using Image J software. To evaluate the apoptosis index of heart TUNEL stained tissues, 10 random heart fields per tissue section were captured at the 400× magnification.

### DHE staining

The heart tissues were embedded in the optimum cutting temperature compound in the ethanol- dry ice and were cut into 8 um- thick sections. Then the sections were infused in DHE (10 μmol/L) in a dark humidified chamber at 37°C for 30 min. Then the sections were detected by fluorescence microscopy.

### Statistical analysis

Values are presented as means ± SD of three independent experiments. Significant differences between all groups were computed by one-way analysis of variance using the Student-Newman-Keuls post hoc test for multiple group comparisons. Statistical difference was accepted at p<0.05.

## Results

### Serum Ad levels

**Figure 1:**
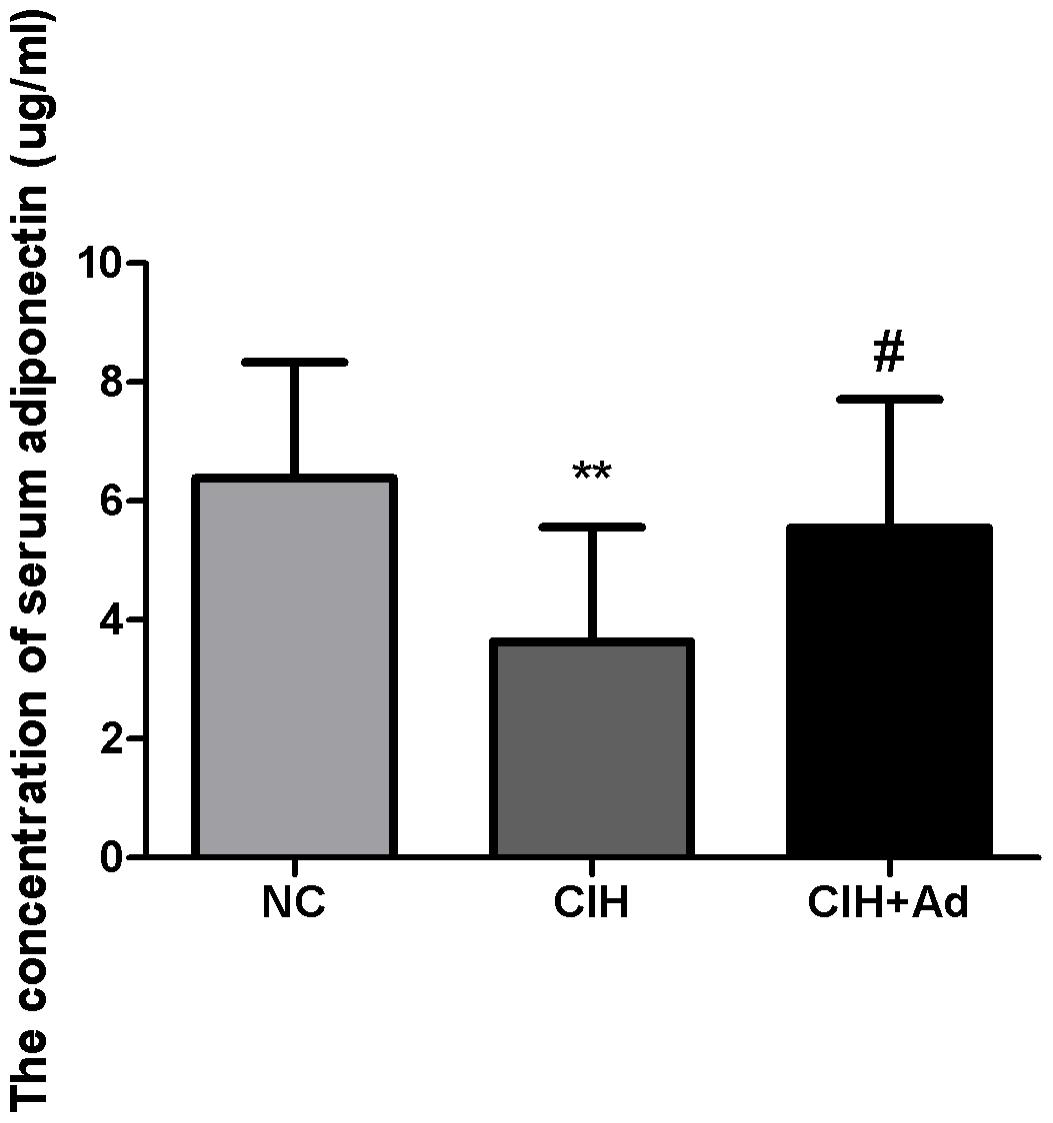
Serum adiponectin levels in the three groups. NC, normal control group; CIH, chronic intermittent hypoxia; CIH + Ad, chronic intermittent hypoxia and adiponectin supplement; **P<0.01 versus NC group; #P<0.05 versus CIH.

Serum Ad level was lower in the CIH group than in the NC group and CIH + Ad group (p<0.05) (Figure 1). There was no significant difference between the NC group and CIH + Ad group in serum Ad level (p>0.05). The results are consistent with those of our previous study [30].

### Echocardiographic Data

Compared with the NC group, the echocardiograph, on day 35 of the experiment, showed that in the CIH group there was a significant elevation in both LVDs (4.197±0.6035 mm vs 3.276±0.6192 mm, p<0.05) and LVESV (77.86±21.18 μl vs 47.60±24.32 μl, p<0.05) along with the expected decrease in both LVEF (67.97±6.687% vs 80.75±7.624%, p<0.01) and LVFS (39.26±5.905% vs 51.42±8.047%, p<0.01) (Table 1). However, compared with the CIH group alone, the left ventricular function parameters mentioned above had improved compared to CIH + Ad group (p<0.05). There were not any noted significant differences in LVEDV, LVDd and HR among the three groups (p>0.05) (Table 1).

**Table 1:** Echocardiographic data —5 weeks.

	NC (n = 15)	CIH (n = 15)	CIH+Ad (n = 15)	*P Value	#P Value
LVDd, mm	6.744±0.4660	6.823±0.438	6.312±0.715	NS	NS
LVDs, mm	3.276±0.6192	4.197±0.6035*	3.330±0.7568#	<0.05	<0.05
LVFS, %	51.42±8.047	39.26±5.905*	47.86±7.370*#	<0.05	<0.05
LVEF, %	80.75±7.624	67.97±6.687*	77.66±7.324*#	<0.05	<0.05
HR, bpm	413.2±52.14	378.3±75,73	376.1±79.88	NS	NS
LVEDV, μl	240.5±36.65	239.7±33.43	203.6±46.59	NS	NS
LVESV, μl	47.60±24.32	77.86±21.18*	47.58±22.35#	<0.05	<0.05

Values are means ± SD; n, number of animals exposed to CIH or room air for 5 weeks; HR, heart rate; LVDd, left ventricular end-diastolic diameter; LVDs, left ventricular end-systolic diameter; LVEDV, left ventricular end-diastolic volume; LVESV, left ventricular end-systolic volume; LVEF%, left ventricular ejection fraction; LVFS%, left ventricular percent fractional shortening. *P<0.05 versus NC group; #p<0.05 versus CIH.

### ER stress

**Figure 2:**
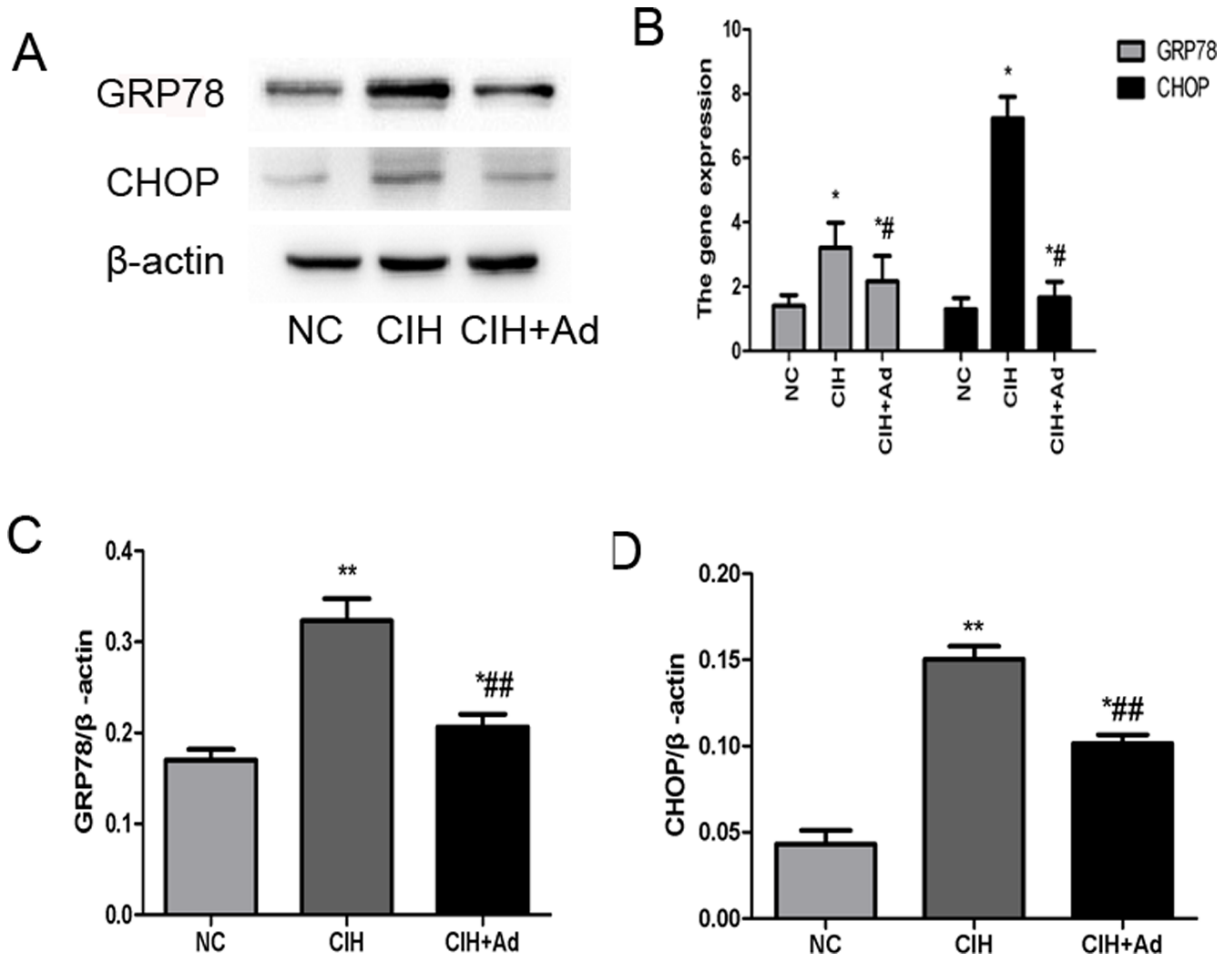
The UPR induction after CIH. A: Western blot analysis of GRP78 and CHOP in whole heart tissue homogenates. WB bands were normalized to β-actin. B: mRNA expressions of GRP78, CHOP in heart of three groups; PCR fluorescent signals for GRP78 and CHOP were standardized to PCR fluorescent signals obtained from an endogenous reference (β-actin). C: the densitometric evaluation of the independent western blot of GRP78; D: the densitometric evaluation of the independent western blot CHOP; *P<0.05 and **P<0.01 versus NC group; #p<0.05 and ##P<0.01 versus CIH group.

**Figure 3:**
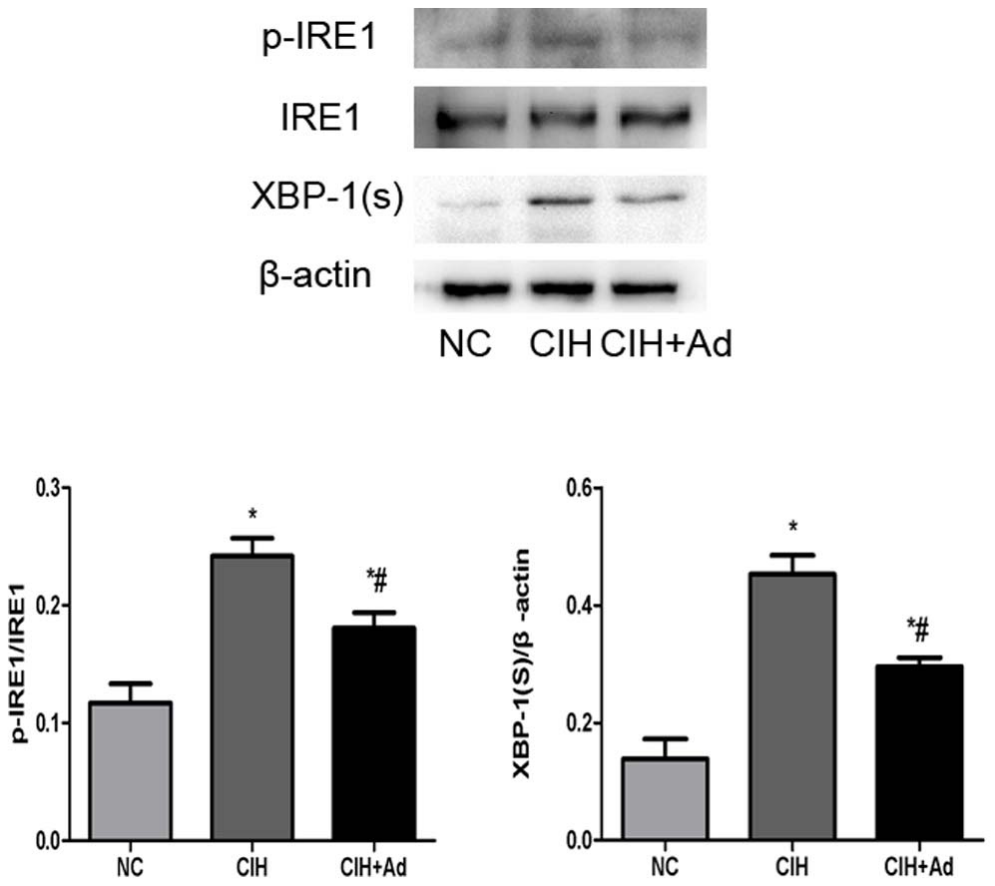
IRE1 pathway activation in heart. The protein levels of p-IRE1, IRE1, XBP-1 (s). Western blot band of p-IRE1 was normalized to IRE1. *P<0.05 versus NC group; #p<0.05 versus CIH.

**Figure 4:**
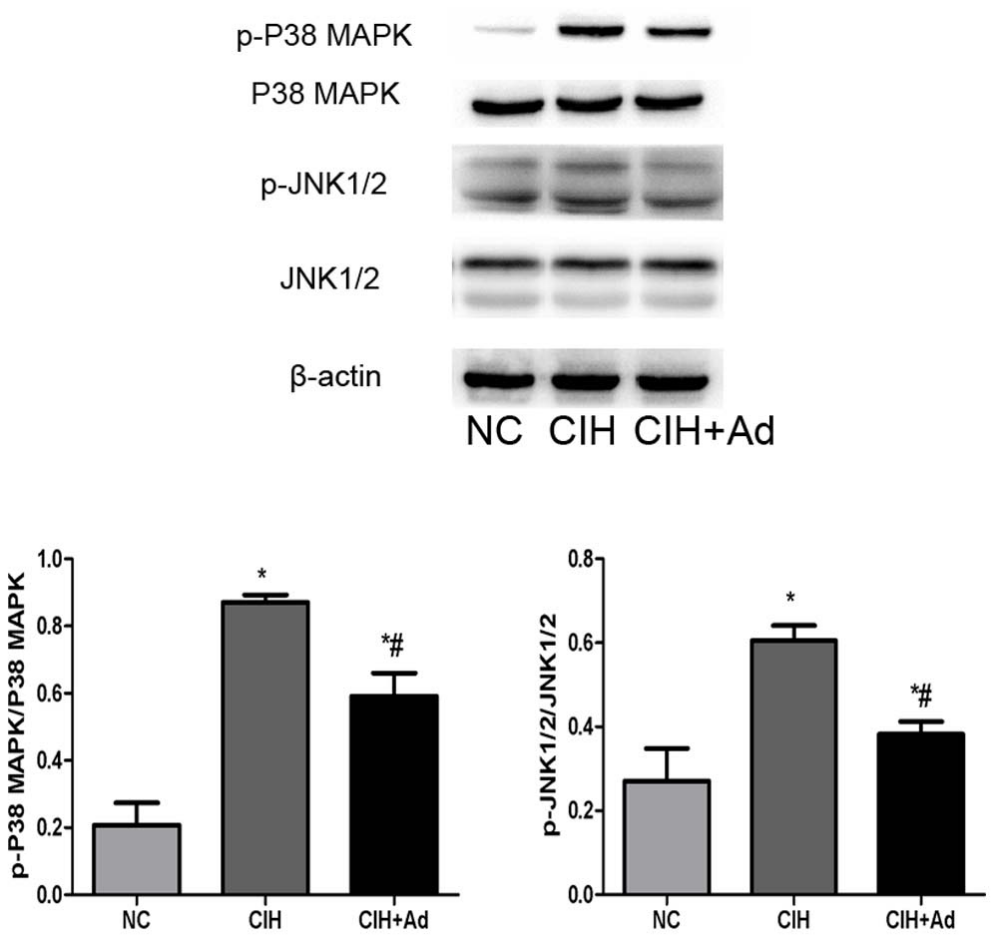
The protein levels of JNK1/2, P38 MAPK in heart. The protein levels of p-JNK1/2, JNK1/2, p-P38 MAPK and P38 MAPK. Western blot bands of p-JNK1/2 and p-P38 MAPK were separately normalized to JNK1/2 and p-P38 MAPK. *P<0.05 versus NC group; #p<0.05 versus CIH group.

**Figure 5:**
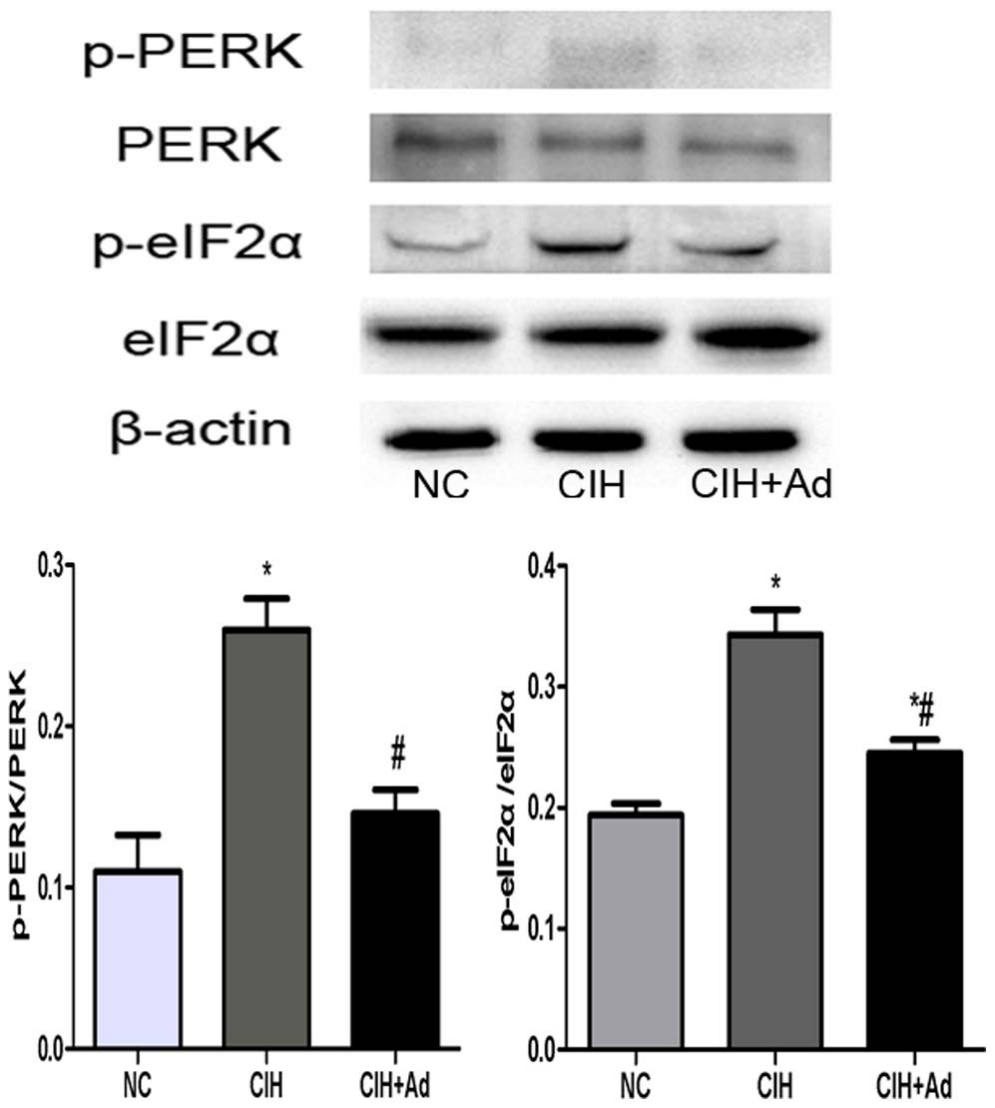
PERK pathway activation in heart. The protein levels of p-PERK, PERK, p-eIF2α, eIF2α. Western blot bands of p-PERK and p-eIF2α were separately normalized to PERK and eIF2α. *P<0.05 versus NC group; #p<0.05 versus CIH group.

**Figure 6:**
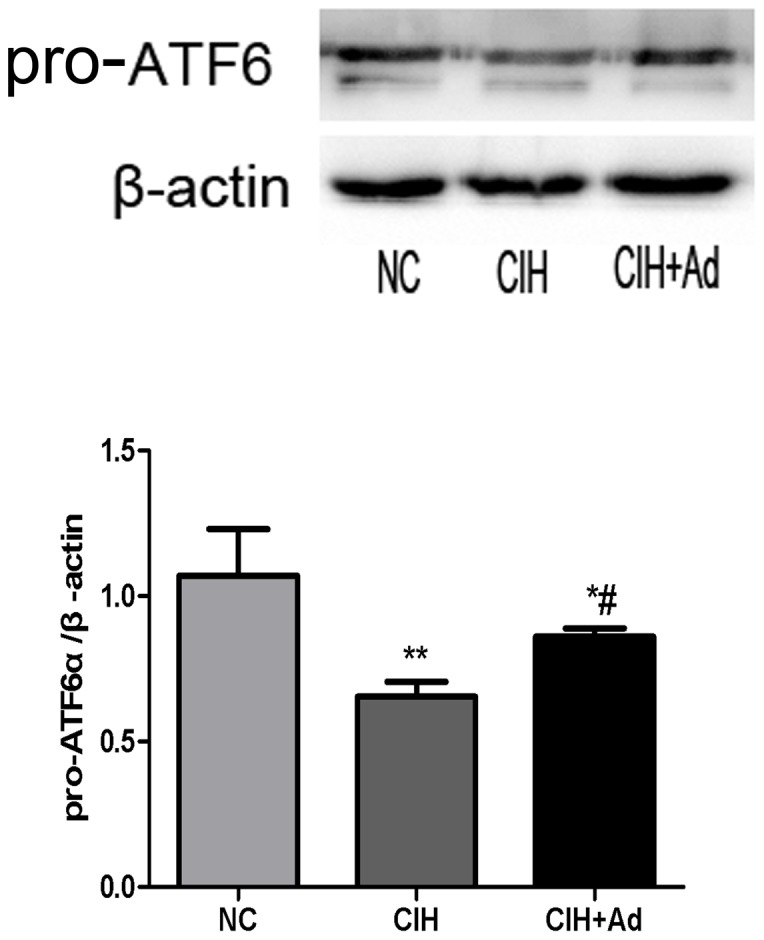
ATF6 pathway activation in heart. The protein levels of pro-ATF6; *P<0.05 versus NC group; #p<0.05 versus CIH group.

In order to evaluate whether CIH could exacerbate ER stress, Western blot and Semi-quantitative real-time PCR were used to explore the gene and protein expression levels of the following two kinds of molecules: GRP78, an ER chaperone, and CHOP, a well studied protein crucial to growth arrest and DNA damage. Both proteins are known to be specifically induced by ER stress. The gene and protein levels of GRP78 and CHOP were significantly enhanced in the CIH group compared to both NC group and CIH + Ad groups (p<0.05) (Figure 2). With increased GRP78 and CHOP protein expression, we examined which of the three major transducers of ER stress were activated after CIH. The protein expression levels of IRE1 and XBP-1(s) were examined to assess for involvement of IRE1 pathway in CIH induced ER stress. In the CIH group, the expression of IRE1 and XBP-1(s) protein levels significantly increased as compared to the NC group and CIH + Ad group (p<0.05), even though there was a statistical difference between the NC group and CIH + Ad group (Figure 3). Activated IRE1 has been located upstream of the phospho-JNK1/2 and phospho-p38 MAPK pathways [31], [32]. We noted a significantly increase in the protein levels of phospho-JNK1/2 and phospho-p38 MAPK in the CIH group as compared with both NC group and CIH + Ad group (p<0.05), although protein levels in the CIH + Ad group were statistically higher than the NC group alone (p<0.05) (Figure 4). Protein levels of phospho-PERK and phospho-eIF2α were significantly different among the three groups (p<0.05), which were the highest in the CIH group but the lowest in the NC group, with intermediate levels in the CIH + Ad group (Figure 5). The protein expression levels of pro-ATF6 were also markedly different among three groups (p<0.05), which were the highest in NC group but the lowest in CIH group, with CIH + Ad group in between (Figure 6).

### ROS

**Figure 7:**
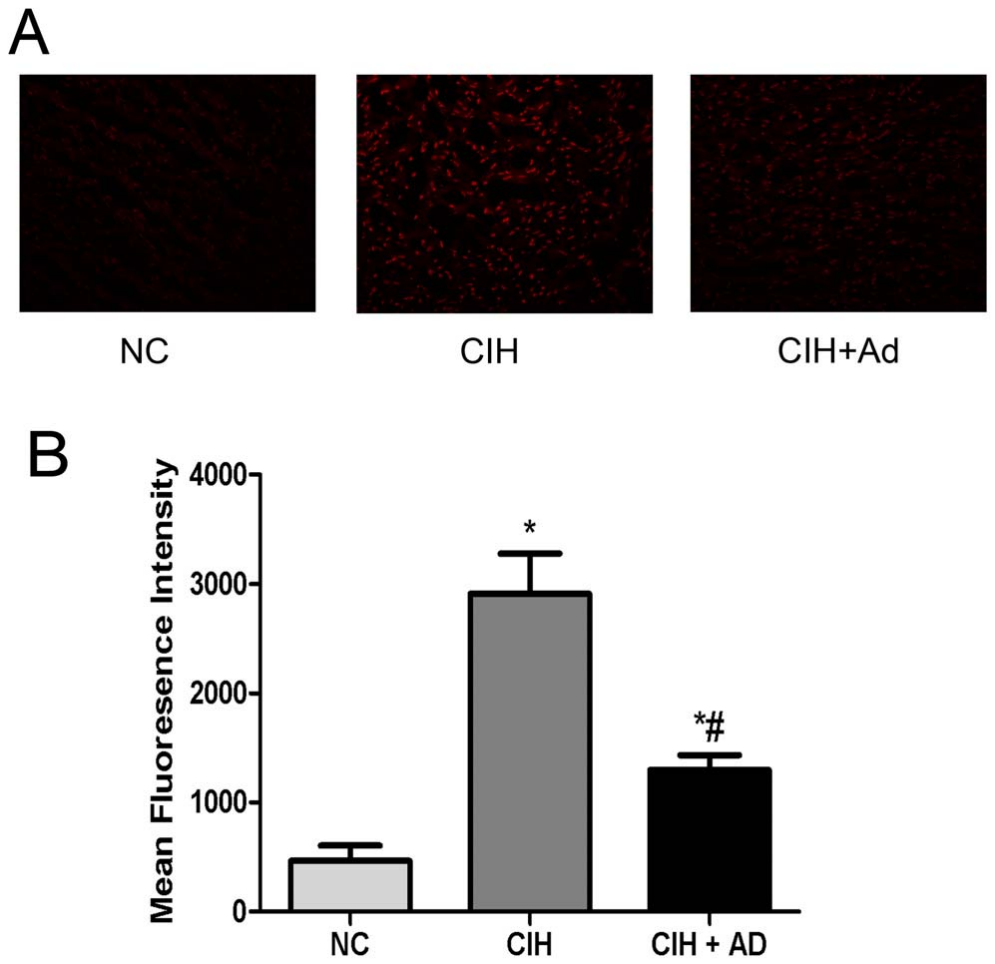
Detection of ROS generation of myocardium in three groups. A: intracellular ROS generation detection in heart tissue. B: the values of fluorescence intensity in three groups. *P<0.05 versus NC group; #p<0.05 versus CIH group.

At the end of the experiment, myocardial reactive oxygen species (ROS) was detected by DHE staining (Figure 7). The intensity of DHE fluorescence in the CIH group showed significantly stronger fluorescent intensity compared to the NC group (p<0.05). As expected, the intensity of DHE fluorescence staining in the NC + Ad group were significantly decreased compared with the CIH group, although significantly higher than that in NC group (p<0.05).

### Myocardial apoptosis

**Figure 8:**
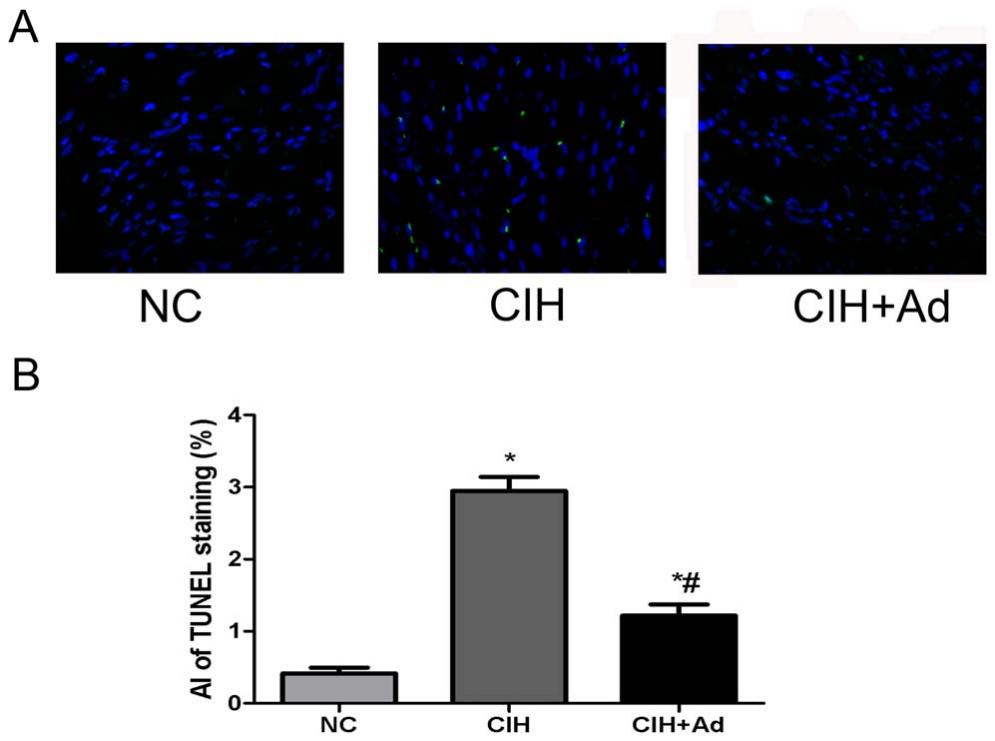
The TUNEL staining of the heart. Nuclei are shown in blue, and TUNEL staining is shown in green. The lower panels show the percentage of TUNEL-positive cell. *P<0.05 versus NC group; #p<0.05 versus CIH group.

**Figure 9:**
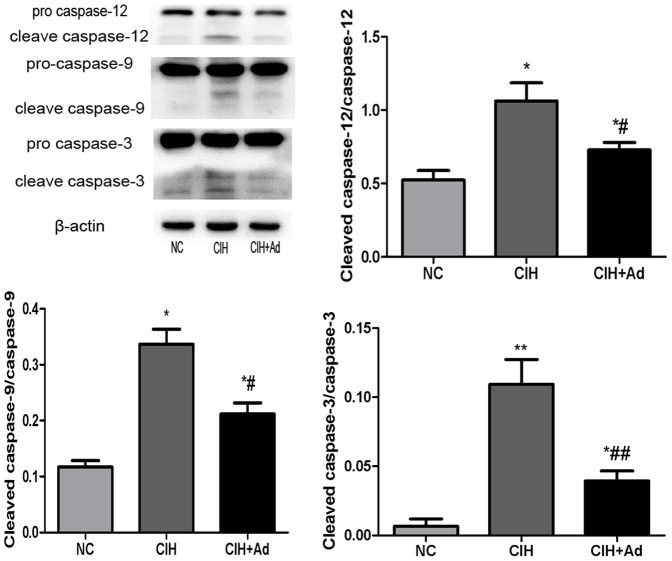
The protein levels of caspase-12, caspase-9, caspase-3. Western blot bands of cleaved caspase-12, cleaved caspase-9 and cleaved caspase-3 were separately normalized to caspase-12, caspase-9 and caspase-3. *P<0.05 and **P<0.01 versus NC group; #p<0.05 and ##P<0.01 versus CIH group.

After 35 days' exposure to CIH, the presence of myocardial cells undergoing apoptosis was determined by TUNEL staining. The percentage of labeled TUNEL-positive cells in myocardium was the highest in the CIH group (2.948±0.1936%) but the lowest in the NC group (0.4167±0.07915%), with the CIH + Ad group (1.219±0.1512%) in between. There was a significant difference among all three groups (p<0.05) (Figure 8). Cleaved caspase-12, a specific molecular marker of apoptosis as a result of ER stress, as well as cleaved caspase-9 and cleaved caspase-3, two important biochemical markers of apoptosis were also tested. The protein levels of the three markers showed significant difference among three groups (p<0.05), which were the highest in CIH group but the lowest in NC group, with CIH + Ad group in between (Figure 9).

## Discussion

By observation the effects of CIH on myocardial function *in vivo*, we found that CIH could induce left ventricular dysfunction as indicated by elevated LVDs and LVESV and reduced LVEF and LVFS in Wistar rats. Enhanced ER stress and ROS with associated apoptosis were our proposed mechanism of myocardial damage and we believed Ad supplementation attenuated the resulting myocardial dysfunction and associated myocardial apoptosis through inhibition of oxidative and ER stress.

It has been reported that OSAS can induce myocardial dysfunction in humans [33]. To explore this mechanism, an animal model of CIH was established in this study. In accordance with our hypothesis, the results showed that left ventricular dysfunction was found in rats exposed to CIH for 5 weeks. Our findings are consistent with the results from Chen et al [29], [34], [35]. Apoptosis was found to be an important mechanism in several types of cardiomyopathies and decompensated human heart tissues whereby attenuating myocardial apoptosis can protect left ventricular function [36], [37], [38]. Conversely, enhancing myocardial apoptosis can intensify cardiac dysfunction [39], [40], [41]. Taken together, we speculated that myocardial apoptosis may play an important role in cardiac dysfunction after CIH exposure. Therefore, in the present study, we detected caspase-3 expression, a critical biochemical marker of apoptosis [42] with TUNEL assay to assess for the presence and relative levels of myocardial apoptosis in our two experimental groups and NC group. The results showed elevated cleaved caspase-3 levels and TUNEL-positive cells after CIH exposure indicative of post-CIH enhanced myocardial apoptosis. However, when Ad was supplemented, both cardiac dysfunction and myocardial apoptosis had partially improved. It is well known that Ad has a cardioprotective effect. It has been reported in Ad deficient mice that there was enhanced myocardial damage and cardiac dysfunction induced by ischemia/reperfusion [43], [44], [45] or pressure overload [20]. It has been reported that Ad protected the myocardium against apoptosis induced by hypoxia/reoxygenation [46]. So we speculated that Ad might improve the cardiac dysfunction by reducing the myocardial apoptosis. In order to study the interplay between the mechanisms of apoptosis and inhibition by Ad, we studied the ER stress.

The endoplasmic reticulum (ER) is an important organelle which modulates protein biosynthesis and folding, lipid biosynthesis, cell homeostasis and apoptosis and calcium homeostasis. Once the homeostasis is broken, accumulation of unfolded and misfolded proteins in the ER led to ER stress [26]. To cope with unfold and misfolded proteins, the ER activates the transcriptional and translational pathways, which is called the unfold protein response (UPR) [26], [47]. The UPR in mammalians has three branches: PERK pathway, IRE1 pathway and ATF6 pathway [48], [49], [50].

Once the injury is excessive, the UPR can induce cell apoptosis [31], [48], [49], [50]. Under excessive ER stress, the phosphorylated IRE1 recruits the TNFR-associated factor 2, which activates the downstream target phospho-JNK and phospho-p38 MAPK [31], [32]. It has been reported that the phosphorylation of JNK activated both pro-apoptotic BIM and inhibited anti-apoptotic BCL-2 [51], members of the BCL-2 family. In addition to IRE1 pathway, the activated PERK pathway and ATF6 pathway are also involved in the ER stress-associated apoptosis. Activated ATF4 and ATF6 can activate CHOP, a specially pro-apoptosis molecular of ER stress, which is also a downstream target of XBP-1 [52]. Several studies reported that the expression of CHOP was linked to the apoptosis induced by ER stress [53], [54], and CHOP deficiency could protect the cell against apoptosis induced by excessive ER stress [55], [56]. CHOP can down-regulate BCL-2 and up-regulate BIM [57]. The BCL-2 related proteins' family is important in regulating apoptosis and they in part represent for the apoptosis [42], [58]. Caspases, a family of cysteine proteases, are involved in apoptosis and certain members are associated with the ER stress [59]. Caspase-12 is specifically localized in ER and plays an important role in ER stress-induced cell death [60]. Activated caspase-12 by means of excessive ER stress, cleaves the procaspase-9 into active caspase-9, which in turn actives pro caspase-3, leading to apoptosis [61]. In the present study, three arms of the UPR were detected first after CIH. The activated UPR demonstrates presence of the ER stress. We also detected the JNK, CHOP and caspase family to evaluate the apoptotic pathway. ER stress associated apoptosis was also enhanced demonstrated by increased expressions of p-JNK, CHOP, cleaved caspased-12, cleaved caspased-3. However, after Ad supplement action was complete, the three arms of UPR and the expressions of p-JNK, CHOP, cleaved caspased-12, cleaved caspased-9 and cleaved caspased-3 were all reduced. So we suggested Ad could inhibit the ER stress and the associated apoptosis. However, how CIH induced ER stress and the underling mechanisms of the ER stress in relation to the inhibition of Ad were also unknown. In order to find out the mechanisms, we further detected ROS in myocardium. It has been reported that ROS is one of the stimuli which can trigger ER stress [62], [63], known as ROS- dependent ER stress. In this study, we found an increased ROS in myocardium of the CIH animals. So we suggested the ER stress may be induced by ROS and supplement of Ad could reduce ROS levels. Goldstein et al has reported that Ad could inhibit ROS production after myocardium ischemia reperfusion injury [10]. Magalang et al has reported that Ad could inhibit ROS generation by neutrophils [64]. Our results are consistent with the current literature, which led us to suggest that Ad inhibits the ER stress by means of ROS suppression.

**Figure 10:**
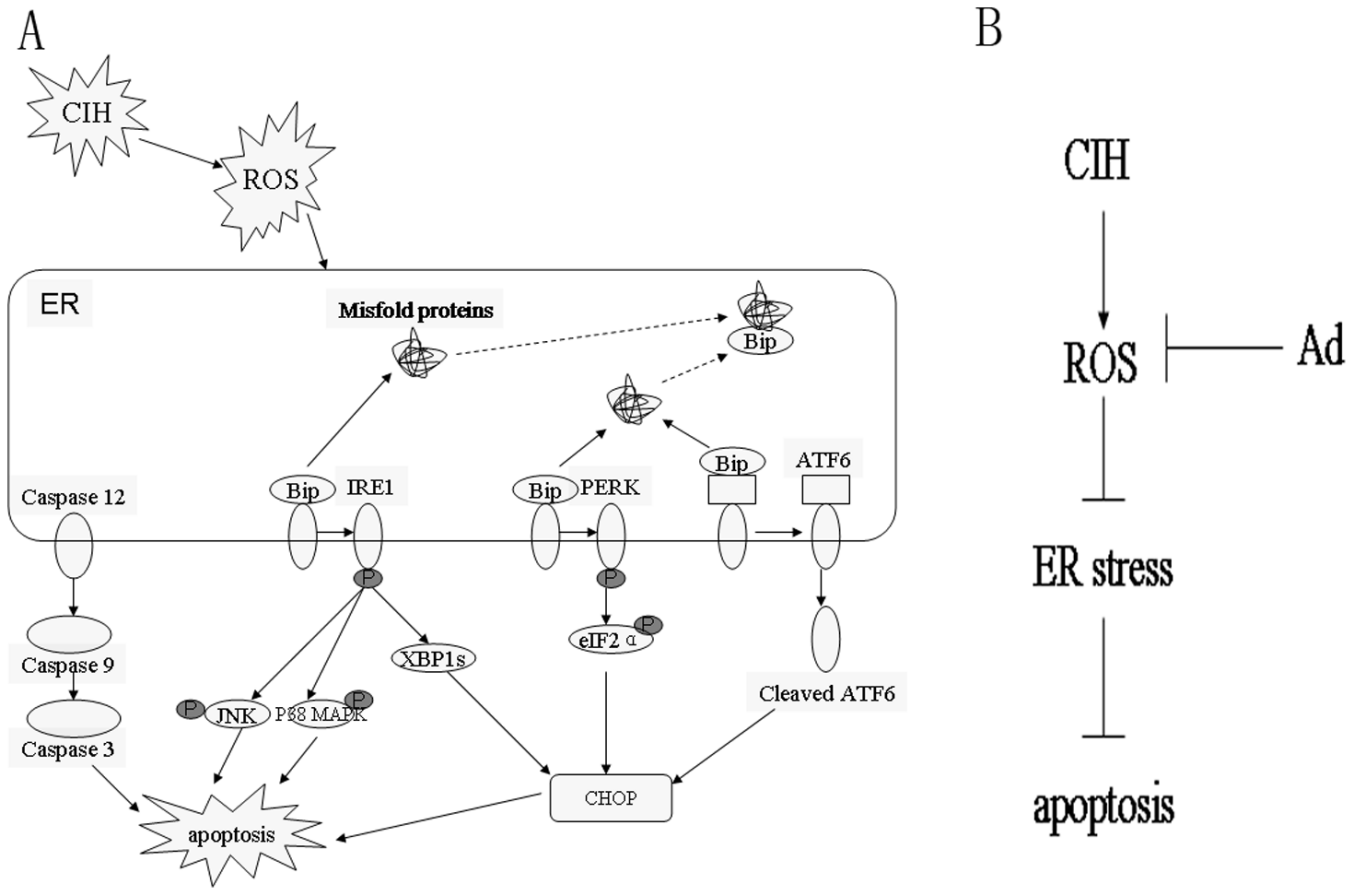
Possible mechanisms of myocardium apoptosis induced by CIH. (A) and protective roles of Ad (B). A: CIH can induce the ROS and then further causes ER stress represented by generation of misfold proteins, which combine Bip released from IRE1, PERK and ATF6. Upon Bip release, IRE1, PERK and ATF6 are activated. All of the three pathways finally upregulate the expression of CHOP, which may further trigger cell apoptosis. The IRE1 pathway also activates the JNK and P38 MAPK, which can trigger apoptosis. In addition, caspase-12 is activated during ER stress, which sequentially activates caspase-9 and/or caspase-3, leading to apoptosis. B: Through inhibition of ROS, Ad supplement may further suppress ERS and therefore the myocardial apoptosis may be reduced.

Overall, as shown in Figure 10, our study establishes the role of Ad as a potential cardioprotective agent against CIH-induced cardiac dysfunction and its resulting myocardial apoptosis possibly by down-regulating ROS-dependent ER stress.
